# The Impact of COVID-19 on Knowledge, Beliefs, and Practices of Ni-Vanuatu Health Workers Regarding Antibiotic Prescribing and Antibiotic Resistance, 2018 and 2022: A Mixed Methods Study

**DOI:** 10.3390/tropicalmed8100477

**Published:** 2023-10-18

**Authors:** Nicola D. Foxlee, Siti Aishah Taleo, Agnes Mathias, Nicola Townell, Lachlan McIver, Colleen L. Lau

**Affiliations:** 1 National Centre for Epidemiology and Public Health, Australian National University, Canberra, ACT 2600, Australia; 2 Dispensary, Vila Central Hospital, Ministry of Health, Private Mail Bag, Port Vila 9009, Vanuatu; 3 Curative Services, Ministry of Health, Private Mail Bag, Port Vila 9009, Vanuatu; 4 Pacific Region Infectious Diseases Association, Kenmore Hills, QLD 4069, Australia; 5 Rocketship Pacific, Estella, NSW 2650, Australia; 6 School of Public Health, Faculty of Medicine, The University of Queensland, Herston, QLD 4006, Australia; colleen.lau@uq.edu.au

**Keywords:** antibiotic prescribing practices, antimicrobial resistance, antibiotic resistance, knowledge and beliefs, antibiotic stewardship, Vanuatu, low- and middle-income countries

## Abstract

Antimicrobial resistance (AMR) is included in the ten most urgent global public health threats. Global evidence suggests that antibiotics were over prescribed during the early waves of the COVID-19 pandemic, particularly in low- and middle-income countries. Inappropriate use of antibiotics drives the emergence and spread of antibiotic resistance. This study aimed to examine the impact of COVID-19 on Ni-Vanuatu health worker knowledge, beliefs, and practices (KBP) regarding antibiotic prescribing and awareness of antibacterial AMR. A mixed methods study was conducted using questionnaires and in-depth interviews in 2018 and 2022. A total of 49 respondents completed both baseline (2018) and follow-up (2022) questionnaires. Knowledge scores about prescribing improved between surveys, although health workers were less confident about some prescribing activities. Respondents identified barriers to optimal hand hygiene performance. More than three-quarters of respondents reported that COVID-19 influenced their prescribing practice and heightened their awareness of ABR: “more careful”, “more aware”, “stricter”, and “need more community awareness”. Recommendations include providing ongoing continuing professional development to improve knowledge, enhance skills, and maintain prescribing competency; formalising antibiotic stewardship and infection, prevention, and control (IPC) programmes to optimise prescribing and IPC practices; and raising community awareness about ABR to support more effective use of medications.

## 1. Introduction

Coronavirus 2019 (COVID-19), caused by the Severe Acute Respiratory Syndrome Coronavirus 2 (SARS-CoV-2) [1], was first detected in Wuhan, China and declared a Public Health Emergency of International Concern by the World Health Organization (WHO) in early 2020 [2]. During the initial stages of the infection, the symptoms, signs, and radiographic changes of COVID-19 overlap those of many other respiratory tract infections, making it challenging to distinguish from illness caused by other pathogens, secondary bacterial infections, or co-infections [3].

Antimicrobial resistance (AMR) was highlighted as a global public health threat in 2014 at the World Health Assembly (WHA) [4]. Research predicts that by 2050, AMR will be responsible for some 10 million deaths annually and cost upwards of USD 100 trillion [3]. Infection prevention and control (IPC) and antibiotic stewardship programs (ASPs) were identified as key strategies to address AMR in healthcare settings in objectives three and four of WHA’s Global Action Plan (GAP) to contain AMR [4]. All members of the WHA agreed to develop and implement a national action plan (NAP) based on the GAP’s five objectives. Given the constraints they face and the added burden of the pandemic, many low- and middle-income-countries (LMICs), like Vanuatu and other Pacific Island Countries and Territories (PICTs), have been slower to achieve this [5].

Evidence indicates that antibiotics were over and unnecessarily prescribed during the first waves of the COVID-19 pandemic [6,7,8,9]. Although this was found to be greater in some LMICs [10,11,12,13], studies on the impact of COVID-19 in LMICs are scarce. Research globally suggests that the prescribing of antibiotics may have been far higher than the prevalence of secondary bacterial infections and co-infections may have been necessitated [11,14,15,16,17]. Increased and inappropriate use of antibiotics are important drivers for the emergence and spread of AMR, including antibacterial resistance (ABR) [3,18]. Some over-prescribing may have been unavoidable during the early stages of the COVID-19 pandemic [19] because of substantial uncertainties about optimal clinical management, lack of treatment protocols, shortages of trained staff, supply line disruptions, and an overwhelming number of patients presenting for treatment [3]. It is likely that LMICs may have been impacted by COVID-19–associated ABR to a greater extent than HICs. In LMICs, awareness about ABR is generally lower, microbiology capacity often limited, and health systems already stretched, so the need and urgency to implement strategies to manage and contain the pandemic may have resulted in further stress to the system [3].

In 2018, development of a national antibiotic guideline commenced and was interrupted by the COVID-19 pandemic. Engaging Ni-Vanuatu health workers in guideline development is pivotal to ensuring uptake and adherence to the guideline. Gaining an understanding of the knowledge, beliefs, and practices (KBP) of Ni-Vanuatu health workers regarding antibiotic-prescribing and awareness of ABR is appropriate and timely and may provide valuable information to inform these efforts. Therefore, the aim of this study was to examine health worker KBP regarding antibiotic prescribing and awareness of ABR and to investigate the impact of the COVID-19 pandemic on health worker KBP with respect to antibiotic prescribing and ABR.

## 2. Material and Methods

### 2.1. Study Design: Mixed Methods Sequential Explanatory Design

This study used a mixed methods design. Data collection included two components: (i) a quantitative KBP questionnaire which included both closed and open-ended questions, and (ii) face-to-face and telephone interviews. The rationale for using a mixed methods design was to provide participants with the opportunity to add clarification and explanation to the quantitative questionnaire responses [20,21].

**Figure 1:**
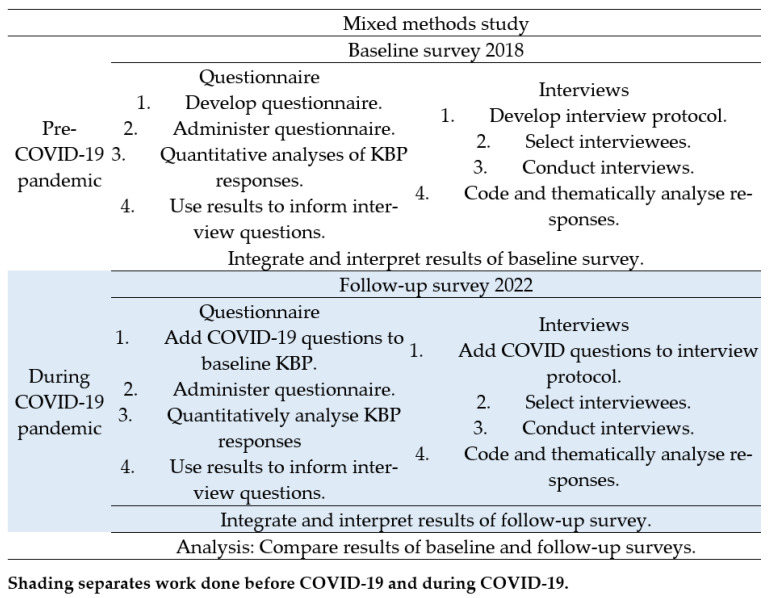
Visual model of mixed methods study into the impact of COVID-19 on knowledge, beliefs, and practices (KBP) regarding antibiotic prescribing and awareness of antibiotic resistance in Vanuatu.

Our mixed methods approach prioritised quantitative over qualitative methodology in a sequential explanatory design [20]. We considered the research question, which method should take precedence, the order that the data collection and analysis should occur, and when the data would be combined [20,22]. More information can be found in Figure 1, which provides a visual model of the mixed methods sequential explanatory design procedures [20].

### 2.2. Study Setting

The Republic of Vanuatu is a PICT with a population of 319,000 [23]. The country is divided into two Health Directorates: (Southern and Northern), each served by a referral hospital, Vila Central Hospital (VCH) and the smaller Northern Provincial Hospital (NPH), respectively. Vanuatu’s main referral hospital, VCH, has 230 beds catering for patients in the paediatrics, medicine, obstetrics and gynaecology, mind care, tuberculosis, and surgery wards. The following outpatient services are provided: emergency; adult; paediatric; surgery; oral health; eye care; maternity and family planning; ear, nose, and throat care; and physiotherapy. There is also a medical laboratory and a radiology unit.

### 2.3. Quantitative Knowledge, Beliefs, and Practices (KBP) Survey

Two validated questionnaires were used to develop the survey instrument: a knowledge, attitudes, and practices survey from the University of New South Wales (UNSW) [24] and the WHO infection prevention and control assessment framework [25]. After permissions were obtained to use the UNSW survey, several questions were selected and adapted for use in our survey. The remaining questions and clinical scenarios were based on the Vanuatu Health Workers Manual (VHWM) [26], the Vanuatu COVID-19 Protocol [27], and the Fiji Antibiotic Treatment Guideline [28]. The baseline KBP questionnaire consisted of 39 questions and five clinical scenarios. The tool covered four areas, including participant demographics, knowledge, and beliefs about antibiotic prescribing, ABR, and prescribing practices. Antibiotic prescribing knowledge was assessed using five clinical scenarios. Each clinical scenario included nine antibiotics and other treatment choices. In order to assess confidence in prescribing skills, health workers were presented with a list of eight statements about activities associated with prescribing antibiotics and asked to indicate their level of confidence with each. Likert scale questions were used to measure knowledge, beliefs, and awareness about ABR. Health worker’s KBPs regarding IPC were evaluated using WHO’s five hand hygiene moments and other questions and statements about IPC practice [25]. Health workers were also asked about the information sources they use to inform their prescribing decisions as well as the possible reasons why patients request antibiotics when they may not be required. The follow-up questionnaire included seven additional questions and four additional clinical scenarios relating to prescribing during the COVID-19 pandemic. The baseline questionnaire was available in print form only, whilst the follow-up questionnaire was also available online.

### 2.4. Validation Process

A group of health professionals, both Ni-Vanuatu and Australian, provided feedback on the questionnaire’s face-validity, and a senior Ni-Vanuatu clinician and an Australian infectious disease physician assessed the content validity. A pilot study was conducted on five Ni-Vanuatu health workers who were given both the participant information sheet (PIS) and questionnaire and invited to provide feedback. The KBP questionnaire was revised based on the feedback. The internal consistency reliability of the questionnaire was evaluated using Cronbach’s alpha [29].

### 2.5. Study Participants

In 2018, healthcare workers registered with the Vanuatu Drugs and Therapeutics Committee to prescribe antibiotics and employed at Vila Central Hospital were invited to participate (an estimate of 75). Participants were able to choose how they completed the questionnaire: by face-to-face interview with a researcher, self-completion of a hard copy questionnaire, or self-completion of an online questionnaire (at follow-up only). A total of 65 healthcare workers responded to the baseline survey in 2018 and 67 to the follow-up in 2022.

All participants were invited to participate in face-to-face interviews following completion of the baseline questionnaire. Seven of the interviewees who agreed to participate in the follow-up questionnaire were invited to be re-interviewed after completing the questionnaire. These interviewees were selected by two of the authors (NDF, AM) based on their professional group representativeness and clinical experience.

### 2.6. Statistical Analysis

#### 2.6.1. Quantitative Data

Descriptive statistics were used to summarise the numeric data. The paired t-test was used to compare means when the data were interval and normally distributed and the non-parametric test, Wilcoxon signed-rank test (WSRT), was used to compare ordinal data. The McNemar test for paired data was used to analyse relationships between categorical variables. The alpha level was set as 0.05 and a p value of ≤0.05 was considered statistically significant [30]. The analysis was conducted using Stata version 15 (Stata Corp., College Station, TX, USA).

#### 2.6.2. Qualitative Data

The constant comparative method (a qualitative research technique) was employed to analyse the responses from the open-ended questions and interviews [20]. Since the purpose of the qualitative component was to expand upon the quantitative results, principal themes were identified a priori and based on the subject content of the questionnaire. Within these themes, information with similar content was grouped, reviewed, re-grouped, and refined into higher levels of sub-themes (similarity and contrast principles) using a coding scheme [20]. Selected results were presented in table form and/or integrated with the text where they supported or provided insight into the quantitative results.

#### 2.6.3. Ethics

The study was approved by the Vanuatu Cultural Council and the Australian National University Human Research Ethics Committee (protocol 2017/056). Those who elected to participate were given the participant information sheet providing details about the study, survey instruments, requirements of being a participant, confidentiality, and use of the data. Written consent was obtained from those who agreed to participate.

## 3. Results

### 3.1. Questionnaire Validation

The Cronbach’s alpha coefficient was 0.8566 for the KBP questions relating to health worker confidence and was 0.8739 for those relating to beliefs, suggesting both sets of questions have a high internal consistency.

### 3.2. Demographic and Professional Profile

Results are reported for the 49 participants (49 pairs) who completed both surveys. They comprised 22 nurses and midwives, 21 doctors and a small mixed group of two managers, two dentists, and two pharmacists. To ensure participants remained anonymous, the nurse manager was assigned to the nurses’ and midwives’ group, totalling 23, and the medical manager, dentists, and pharmacists to the doctors’ group, totalling 26. Likewise, since a few doctors were trained in China as opposed to Cuba and the number of respondents in both groups was small, the respondents were grouped together to ensure anonymity. Overall, 47% (23) of participants were female. Of the nurses’ group, 61% (14) were female and aged 45 years and older. In contrast, 65% (17) of the doctors’ group were male, and 46% (12) were aged 35 years and younger. Table 1 provides additional demographic details of the study participants who completed both baseline and follow-up surveys.

### 3.3. Knowledge about Antibiotic Prescribing and Antimicrobial Resistance

#### 3.3.1. Clinical Scenarios

Table 2 provides the number and proportion of respondents who selected correct treatments for each of the five clinical scenarios listed on the KBP questionnaire. Table S1 (supplementary file) provides the four clinical scenarios added to the follow-up KAP questionnaire.

Health workers’ clinical prescribing knowledge was examined. The number of correctly selected antibiotics for each clinical scenario by the professional group were recorded and overall, the baseline compared with follow-up can be found in Table 3. Both professional groups showed a significant improvement in surveys. The difference between total baseline and follow-up mean knowledge scores for all health workers was 1.14 *p* < 0.001 (95% CI 0.65; 1.62).

#### 3.3.2. Pathology Services

Participant’s KBP about microbiology laboratory services were examined in questions and statements about the services offered, selecting specimens for testing, and training attended. Table 4 shows the proportion of respondents who provided positive and/or correct responses to the questions and statements. The differences between the baseline and follow-up questionnaire results are provided.

#### 3.3.3. Interview Responses for Pathology Questions

The face-to-face interview responses revealed health worker concerns about laboratory testing. A doctor reported in a baseline interview, “I think some of the cultures we sent were contaminants, like most of the results we got back. We go backwards. It takes you back to the table”. Timeliness was also a concern amongst some interviewees. Another interviewee reported also at baseline, “sometimes the result takes a week” and another respondent added, “upgrade the lab to be timelier”. A participant requested in a follow-up interview that the antibiogram be updated: “updated antibiogram would help”.

### 3.4. Beliefs about Prescribing Antibiotics and Antibiotic Resistance

#### 3.4.1. Confidence in Prescribing Antibiotics

Health worker confidence in prescribing antibiotics was also examined. Table 5. Shows the number of affirmative responses indicating confidence in undertaking the prescribing activities (8) included on the KBP questionnaire. A comparison of the differences between mean scores achieved baseline and follow-up are provided. Table S2 (supplementary file) provides the eight statements listed on the KAP questionnaire used to assess health worker’s antibiotic prescribing confidence.

When comparing the baseline and follow-up confidence scores for all respondents across the eight statements, the findings indicated that health workers were significantly less confident in performing the prescribing activities during the pandemic. The difference between baseline and follow-up mean scores for all health workers was −3.77 *p* < 0.001 (95% CI −1.97; −5.57). There was no correlation in the linear relationship between knowledge and confidence scores r(47) = −0.13, *p* 0.34 [28].

#### 3.4.2. Knowledge, Beliefs, and Awareness of Antibiotic Resistance

Table 6 shows the questions that examined health worker awareness of ABR listed on the KBP questionnaire. The number and proportion of respondents and their level of agreement are presented at baseline and follow-up and compared.

### 3.5. Practices Regarding Hand Hygiene, Prescribing Antibiotics, and Awareness of Antibiotic Resistance

#### 3.5.1. Infection, Prevention, and Control and Hand Hygiene

Questions and statements about IPC and hand hygiene were included on the KBP questionnaire and Table 6 and Table 8 (below in Section 3.5.2) provide the results of the responses to them. 

Participants were also asked in an open-ended question if there were factors which may prevent hand hygiene being performed when hand hygiene was required. There were 25 (51%) and 27 (55%) respondents in the baseline and follow-up questionnaires, respectively, who reported in the affirmative. The results were classified into two groups: lack of supplies and equipment and workload pressures. Table 7 provides the factors reported by the respondents on the baseline and follow-up KBP questionnaires.

#### 3.5.2. Antibiotic Prescribing Practices

Table 8 shows the questions and statements listed on the KAP questionnaire about activities related to prescribing, hand hygiene, and the information sources that are used to inform decision-making by the proportion of respondents and their level of agreement. Table S3 (supplementary file) lists reasons why patients may ask for antibiotics when an antibiotic may not be required and the proportion of respondents who selected the reason. Table S4 (supplementary file) shows a list of statements/questions about prescribing, ABR, and training during COVID-19 and the proportion of respondents by professional group who selected the reason.

At the follow-up, respondents selected the following reasons why they believe patients request antibiotics when an antibiotic is not needed: “perceives antibiotics will make them better when sick” (42; 82%); “travel to hospital is a hardship, so patient asks, just in case” (38; 78%); “unaware of the risk” (32; 76%). Thirteen (13; 26%) respondents selected “patient anxiety due to COVID-19” and 27 (55%), “patient needs reassurance when an antibiotic is unnecessary”. The following participant interview responses supported and clarified these selections:

“Antibiotics most used treatment medicine in all health facilities. People may think antibiotics will heal them when they take it, no matter the problem.”

“If the patient comes from far away then she asks for it just in case she is sick again and it is difficult for her to come back to the hospital for medicine. She lives far and does not have the bus fare.”

“Patients have little knowledge about antibiotics but psychologically they said taking antibiotics makes them feel better.”

“Patients are unaware of antibiotic resistance; they do not have proper consultations with the doctor, and they want to take antibiotics home to store at home for future illness and for family.”

#### 3.5.3. Information Sources

**Figure 2:**
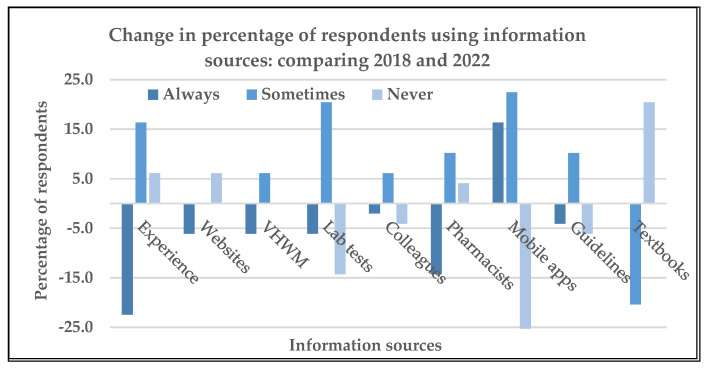
Change in percentage of respondents using the information sources included on the knowledge, beliefs, and practices (KBP) questionnaire about Ni-Vanuatu health workers’ KBP regarding antibiotic prescribing and antibiotic resistance, and the influence of the COVID-19 pandemic: 2018 compared with 2022.

Although usage of information sources to assist with prescribing decisions varied across health workers and between the two questionnaires, the usage of apps on a mobile device was the only tool that obtained significantly higher scores at follow-up compared with the baseline (z = −2.97 *p* < 0.002). Figure 2 shows the change in percentage of respondents using the various information sources in 2018 compared with those in 2022 listed on the baseline KBP questionnaire. Other sources reported included: “Frank Shann’s Drug Doses”, “information on flow chart algorithms”, “discussions for better antibiotic treatment with clinicians”, and “Allergies and beliefs of the patients”.

#### 3.5.4. Influence of COVID-19 Pandemic on Antibiotic Prescribing and ABR

Thirty-seven (37; 72%) health workers correctly answered the four additional COVID-19–related clinical scenarios. Further information can be found in Tables S1 and S4 (supplementary file), which provide the proportions of health workers who selected the correct antibiotic options for the additional clinical scenarios and agreed with the additional statements/questions listed on the follow-up KBP questionnaire, respectively.

The follow-up questionnaire included an open-ended question that asked health workers whether they thought the COVID-19 pandemic had changed their antibiotic prescribing practice and awareness of ABR and if so, in what way. Sixteen nurses (70%) and 22 (85%) doctors responded in the affirmative. Table 9 provides a representative sample of responses with interpretation.

The following responses were provided by two interviewees when asked whether the pandemic had influenced his antibiotic prescribing and/or awareness of ABR:

A senior doctor reported,

“definitely, and this is because of COVID. COVID has made us really more active when it comes to hand hygiene, hygiene, physical distancing and stuff. AMR is just so much more important. … staff at the hospital are right now more into awareness of antibiotic stewardship. Yes. We are a bit more careful with our choice of antibiotics. … There is a noticeable change in our first line antibiotics….”.

A senior nurse reported,

“Yes. Two years ago, we have hygiene and infection control in the clinic, but it is not good, but now after COVID we have to maintain infection control, we wear masks every day, we wash our hands every now and then when we come in contact with a patient, or between ourselves and colleagues. Also, our surfaces are cleaned enough after each patient compared with two years ago.”

## 4. Discussion

The results of this study suggest the COVID-19 pandemic influenced antibiotic prescribing and heightened awareness of ABR amongst Ni-Vanuatu health workers.

Knowledge about antibiotic prescribing improved across both professional groups. There are several possible explanations for this improvement: time between the surveys will have enabled health workers to gain more experience and increase their prescribing competency, and discussion amongst colleagues about different treatment regimens has been shown to improve prescribing practice [31]; and pandemic preparedness included “inhouse” training about treatment planning for viral and bacterial infections [32]. Further, responses from interviewees and open-ended questions support the hypothesis that prescribers believed the pandemic may have improved their antibiotic prescribing practices, “changed our prescribing”, “stricter”, “more careful”, ‘’over-emphasising counselling for viral infections now as they do not need to be treated with antibiotics”.

Health workers’ confidence in performing antibiotic prescribing activities during COVID-19 was shown to be less than before the pandemic.

This uncertainty may be less about actual antibiotic prescribing and more a reflection of how health workers were feeling about the impact of the pandemic. Even though Vanuatu had just seven reported cases of COVID-19 until the borders re-opened in mid-2022 [33], health workers may have been feeling stressed and apprehensive at the effect the global pandemic was having on their already stretched health system, limited staff and equipment, and the impending re-opening of the country’s borders. Health workers may also have been concerned about the challenges of managing sick COVID-19 patients given their situation.

Three-quarters of respondents at follow-up reported using bacterial culture results, either always or sometimes when prescribing antibiotics. Although this is a good result, specimen collection and handling, ordering the correct test, and interpreting the result cannot be assumed to be optimal in all cases. A recent study from Ethiopia (2021) stressed the need to use standardised clinical specimen collection procedures for microbiology and accurate diagnosis and treatment [34]. The same study reported that many LMICs lack standardised procedures [34]. Our study suggests that selecting the correct specimens for testing based on clinical presentation may be challenging for some health workers, suggesting that a better understanding of the microbiology is needed to ensure optimal use of the laboratory. Ferguson et al. report proper specimen selection and collection are often challenging in PICTs [35], providing an explanation for the earlier reference to contamination of pre-analytical samples. The findings of an earlier study support this interviewee’s response [36]. Given the results mentioned above, an ASP educational strategy targeting these skills and including interpretation of culture and antibiotic susceptibility results and cumulative antibiograms would enhance prescriber competencies in these areas and ensure appropriate diagnosis and optimal treatment [37].

Although health workers were aware that ABR was a global issue in 2018, their knowledge about ABR in the PICTs and in Vanuatu was limited and did not change during the survey. Prior to 2018, ABR in Vanuatu had not been investigated, though ABR was present in several PICTs [38,39,40]. By the time COVID-19 had emerged, there was evidence of ABR in Vanuatu, although more work needs to be conducted to build a more “complete picture” [36]. The existence of ABR in PICTs is concerning for Vanuatu. Vanuatu’s rate of methicillin-resistant *Staphylococcus aureus* (MRSA) is just 3% (13/502) [36], whilst in other PICTs, rates range from between >20% and 60% [35,39,41]. Since tourism is a major source of income for Vanuatu and travel between PICTs is popular, the likelihood of resistant bacteria being introduced by travellers, including other Pacific islanders, presents a very real threat for Vanuatu [42].

Despite findings indicating that health workers have a good knowledge of ABR, this does do not necessarily translate into reducing antibiotic prescribing [43,44]. There are factors which, if not addressed, will hinder progress toward achieving optimal prescribing and containment of ABR, including: lack of ongoing CPD for health workers, absence of or nonadherence to antibiotic guidelines, poor IPC in healthcare settings, and lack of community awareness of ABR [3].

A desire to attend “talkings”, “refresher courses”, and “workshops to inform” was indicated by a high proportion of respondents, suggesting an unmet need for professional education experiences. Health worker engagement in continuous professional development (CPD) is crucial to improving and extending knowledge, enhancing skills, and maintaining prescribing competency [45,46]. The importance of CPD in LMICs where trained staff are in short supply cannot be underestimated [47].

Infection prevention and control is one of the five objectives making up the WHA’s GAP to contain AMR [4]. In the early 2000s, the WHO recognised hand hygiene within IPC as the most effective strategy to prevent and control HCAIs [25]. Studies have shown that optimal performance of hand hygiene can achieve a reduction in HCAIs of between 15% and 30% [48,49]. The value of hand hygiene was highlighted during the COVID-19 pandemic [50].

Although there was an improvement in health worker knowledge about IPC, this did not reflect actual hand hygiene practice. The qualitative findings identified obstacles to optimal performance at both baseline and follow-up, including a lack of accessible washing stations, gaps in supplies of soap and hand sanitiser, and lack of time. To ensure adherence to hand hygiene recommendations, the necessary equipment and supplies need to be consistently available at the point-of-care [51]. This may be beyond the capacity of many health systems in LMICs with already stretched budgets. Like many LMIC, Vanuatu is frequently impacted by natural disasters and the COVID-19 pandemic will have been an added burden on the country’s financial resources.

More than a third of patients requested antibiotics when they may not have been needed. A study from Jordan found a similar result (41%) [52]. A study from Nepal reported that nearly half of patients were given antibiotics when not required. Respondents (medical practitioners) attributed this to patient pressure [53]. Study clinicians spoke of “giving in” to patient expectations knowing the condition was unlikely to be bacterial [53]. An Egyptian study confirmed these findings, citing the only factor significantly associated with the receipt of an antibiotic was the patient or caregiver’s preference that one be prescribed (95.2% (59/82 patients) (*p* < 0.05) and 88.6% (109/143 caregivers) (*p* < 0.01)) [54].

Just half of the respondents believed patients who ask for antibiotics need reassurance when an antibiotic is not required. Research into prescriber’s perceptions of a patient’s reasons for requesting antibiotics and the patient’s reasons for doing so is scarce but may clarify misunderstandings [55]. Providing opportunities for health workers to develop communication skills to build relationships and trust with patients has been shown to improve medication adherence and reduce prescribing probability (−6.5% (95% CI −10.7%; −2.3%)) [56]. Further, research has demonstrated that simply explaining why antibiotics are not needed and providing positive treatment advice with an alternate plan for the illness results in reducing unnecessary antibiotic prescribing [57].

In the absence of a national antibiotic guideline, health workers used a wide variety of tools to help them with their treatment decision-making; some may not have been relevant or current. However, in December 2022, Vanuatu’s National Antibiotic Guideline—2022 was released on the Therapeutic Guidelines’ [58] Guideline Host, an online platform which hosts locally developed and endorsed guidelines and protocols for low-resource settings [59]. Ni-Vanuatu prescribers can access the guideline on a mobile device either online or offline when internet access is unreliable. Studies indicate that guidelines that are easy to access have a greater likelihood of being taken up and adhered to [60]. Amongst the respondents, access to mobile devices and the use of online applications was shown to increase significantly between surveys. However, having a guideline available does not guarantee it will be adopted or the recommendations adhered to. An ASP strategy to actively implement, disseminate, monitor, and evaluate the guideline is critical. Engaging stakeholders in each of these processes will engender support and strengthen uptake and compliance [61,62,63].

These authors were unable to identify any studies that investigated the influence of COVID-19 on health workers’ KBP regarding prescribing and awareness of AMR.

The responses to open-ended and interview questions re-affirmed the quantitative results by demonstrating that the majority of participants believe the pandemic influenced their prescribing practices for the better and heightened their awareness of ABR. However, it needs to be noted that the time between surveys (3 years) and other factors may have also contributed to the observed differences between the baseline and follow-up results.

One of the major strengths of this study was the mixed methods design. Employing both quantitative and qualitative research techniques in a study can strengthen the quantitative results [19]. By combining these two techniques, a deeper comprehension of the influence of COVID-19 on Ni-Vanuatu health worker’s KBP regarding antibiotic prescribing and awareness of ABR was gained than by using quantitative techniques alone. The authors believe that this is the first study of its kind to be conducted in Vanuatu and in the PICTs to date.

Even though the study was conducted in the main referral hospital, VCH, the Ni-Vanuatu clinicians move between hospitals when required to cover staff shortages. Although the results cannot be generalised beyond this healthcare setting in Vanuatu, the study may have relevance to similar settings in other PICTs.

Surveys are subject to social bias (acceptable/preferable answers), recall bias on the part of the participants, and interviewer bias from the way questions are asked or responses are received in interviews, and these cannot be ruled out in our study. The open-ended and interview questions did not cover the full range of areas that made up the questionnaire. Therefore, not all the quantitative results had the benefit of added insight from the qualitative research.

## 5. Future Directions

The study found that knowledge about antibiotic prescribing and ABR improved between surveys and some of their beliefs about prescribing and ABR also changed. Together, these may have influenced their practices. The study highlights some of the challenges that may compromise the performance of prescribers in LMICs.

The following recommendations in support of healthcare workers are suggested: providing ongoing CPD activities to improve knowledge, enhance skills, and maintain competency in antibiotic prescribing; formalising antibiotic stewardship and IPC programmes to optimise prescribing and IPC practices; strengthening relationships between prescribers and patients through communication skills training; and raising community awareness about ABR to support more effective use of medications. These are all age-old guidelines for healthcare workers. Moreover, antibiotic stewardship and IPC strategies are integrated into national hospital accreditation standards for hospitals in high-income countries and are being adopted in many middle-income countries [64]. The National Accreditation Board for Hospitals and Healthcare Providers for hospitals in India is an example [65].

Finally, the implementation and dissemination of a new national antibiotic guideline calls attention to the importance of monitoring and assessing its use, and ensuring the guideline maintains currency. These will encourage uptake and adherence to the recommendations and inform ASP strategies to promote the best practice in prescribing.

It is hoped that the results of this study can be translatable to other PICTs and can be used to inform ASP strategies across the region.

## Figures and Tables

**Table 1:** Demographic details at follow-up (2022) of participants who completed both baseline and follow-up surveys about the influence of COVID-19 on Ni-Vanuatu health worker’s knowledge, beliefs, and practices regarding antibiotic prescribing and antibiotic resistance.

Demographics	Nurses, Midwives	Doctors, Dentists, Pharmacists	All Health Workers
	*n* = 23	*n* = 26	*n* = 49
Total participants	*n* (%)	*n* (%)	*n* (%)
Gender			
Male	9 (39)	17 (65)	26 (53)
Female	14 (61)	9 (35)	23 (47)
Age			
≤25 yrs	(0)	(0)	(0)
26–35 yrs	4 (17)	12 (46)	16 (32)
36–45 yrs	7 (31)	8 (33)	15 (31)
>45 yrs	12 (52)	6 (23)	18 (37)
Countries trained			
PNG/Solomon Islands	(0)	4 (15)	4 (8)
Fiji	1 (4)	15 (58)	16 (33)
China/Cuba	(0)	4 (15)	4 (8)
Vanuatu	21 (91)	(0)	21 (43)
Australia/New Zealand	1 (5)	3 (12)	4 (8)
Work experience			
0–3 yrs	(0)	(0)	(0)
3–5 yrs	1 (4)	7 (29)	8 (16)
6–10 yrs	6 (26)	8 (33)	14 (29)
>10 yrs	16 (70)	11 (38)	27 (55)
Work area			
Emergency	4 (17)	5 (19)	9 (18)
Outpatient clinics	7 (29)	2 (7)	9 (18)
Medical with ICU	3 (13)	4 (15)	7 (14)
Surgery and Theatre	3 (13)	5 (19)	8 (16)
Paediatrics with NICU	3 (13)	4 (15)	7 (14)
Obstetrics and Gynaecology	1 (5)	1 (4)	2 (4)
Pharmacy and Dental	1 (5)	4 (15)	5 (10)
Management	1 (5)	1 (4)	2 (4)

**Table 2:** Number and proportion of respondents * who selected the correct antibiotic or other treatment for each of the five clinical scenarios included in the knowledge, beliefs, and practices (KBP) questionnaire about Ni-Vanuatu health worker’s antibiotic prescribing and antibiotic resistance and the influence of the COVID-19 pandemic. The differences in mean scores between baseline (2018) and follow-up (2022) are provided.

Respondents	Nurses, Midwives				Doctors, Dentists, Pharmacists				All Health-Workers			
	*n* = 23		Paired *t* Test		*n* = 26		Paired *t* Test		*n* = 49		Paired *t* Test	
	Baseline *n* (%)	Follow-Up *n* (%)	Mean	*p* Value	Baseline *n* (%)	Follow-Up *n* (%)	Mean	*p* Value	Baseline *n* (%)	Follow-Up *n* (%)	Mean	*p* Value
Clinical case scenarios: Which antibiotic would you recommend?												
1. A mother comes to the emergency with a 4 yr old who has had a fever (39 °C), coryzal discharge, and sore throat for the past 3 days. Otherwise well.												
19(86)		21(96)	0.08	0.42	21 (81)	26(100)	0.19	**0.02**	40 (82)	47 (96)	0.14	**0.03**
2. A 29 yr old pregnant woman (12 weeks) presents without medical history. She complains of having dysuria and fever (38 °C) for the past day.												
5 (22)		12 (52)	1.39	0.08	10 (38)	15 (54)	0.84	0.23	15 (31)	27 (55)	1.1	**0.03**
3. A healthy patient with a 3 cm boil on his leg comes into the outpatient department.												
15 (65)		17 (74)	0.21	0.57	23 (88)	23 (88)	0.53	**0.01**	38 (78)	40 (82)	0.38	**0.06**
4. A 20 yr old woman is admitted with fever, headache, photophobia, and vomiting. On examination, she has a high fever (40 °C) and neck stiffness. A provisional diagnosis of meningitis is given.												
6 (26)		18 (78)	1.86	**0.02**	18 (69)	21 (81)	1.0	0.17	24 (49)	39 (80)	1.41	**0.009**
5. A 30 yr old male comes into emergency with high fever (39 °C) and abdominal pain (5 days). On examination appears drowsy; his blood pressure is 100/60 with pulse of 64 per minute. A provisional diagnosis of typhoid fever is given.												
7 (30)		16 (70)	0.95	0.24	13 (50)	22 (85)	2.38	**0.001**	20 (41)	38 (78)	1.71	**0.002**

Bold = *p* value ≤ 0.05; * Results refer to those providing affirmative and/or correct responses.

**Table 3:** Number of correct antibiotics selected by number of respondents according to professional group for the clinical scenarios listed on the knowledge, beliefs, and practices questionnaire about Ni-Vanuatu health workers’ antibiotic prescribing and antibiotic resistance and the influence of the COVID-19 pandemic. The differences in mean knowledge scores between baseline (2018) and follow-up (2022) results are provided.

	Nurses, Midwives		Doctors, Dentists, Pharmacists		All Health Workers	
	*n* = 23		*n* = 26		*n* = 49	
Number of Correct Antibiotics Selected out of 5 Clinical Scenarios	Baseline *n* (%)	Follow-Up *n* (%)	Baseline *n* (%)	Follow-Up *n* (%)	Baseline *n* (%)	Follow-Up *n* (%)
0	1(4)	0 (0)	0 (0)	0 (0)	1 (2)	0 (0)
1	4 (17)	2 (9)	3 (12)	2 (8)	7 (14)	4 (8)
2	6 (26)	5 (22)	3 (12)	1 (4)	9 (18)	6 (12)
3	7 (31)	6 (26)	5 (19)	0 (0)	12 (24)	6 (12)
4	5 (22)	10 (43)	5 (19)	9 (34)	10 (21)	19 (39)
5	0 (0)	0 (0)	10 (38)	14 (54)	10 (21)	14 (29)
Total number of possible correct answers = *n*	*n* = 115		*n* = 130		*n* = 245	
Number and proportion Correct = *n* (%)	67 (58)	70 (60)	94 (72)	110 (86)	165 (63)	180 (73)
Differences between mean knowledge scores baseline and follow-up; *p* value (95% CI) *	1.56; *p* < **0.001** (0.91; 2.21)		0.76; *p* **0.03** (0.05; 1.48)		1.14 *p <* **0.001** (0.65; 1.62)	

Bold = *p* value ≤ 0.05; * Paired *t* test.

**Table 4:** Number and proportion of respondents * who gave positive and/or correct answers to questions about pathology services included on the knowledge, beliefs, and practices questionnaire about Ni-Vanuatu health workers’ antibiotic prescribing and antibiotic resistance and the influence of the COVID-19 pandemic. The differences between baseline (2018) and follow-up (2022) results are provided.

	Nurses, Midwives				Doctors, Dentists, Pharmacists				All Health Workers			
Respondents	*n* = 23		McNemar Test		*n* = 26		McNemar Test		*n* = 49		McNemar Test	
	Baseline *n* (%)	Follow-Up *n* (%)	X^2^	*p* Value	Baseline *n* (%)	Follow-Up *n* (%)	X^2^	*p* Value	Baseline *n* (%)	Follow-Up *n* (%)	X^2^	*p* Value
1. Does the laboratory offer bacterial culture results and antibiotic susceptibility testing?												
Yes	19 (93)	20 (87)	0.14	0.71	25 (96)	26 (100)	1	0.31	44 (90)	46 (94)	0.05	0.47
2. Correctly identified the important specimens to send for microbiology testing.												
Yes	11 (48)	13 (61)	0.18	0.66	17 (57)	18 (69)	0.06	0.81	26 (53)	31 (63)	0.61	0.43
3. Do you trust in the microbiology test results?												
Yes	18 (78)	22 (96)	1.8	0.17	24 (92)	26 (100)	2.0	0.15	42 (86)	48 (98)	4.5	**0.03**
4. Do you agree that local antibiotic resistance patterns may inform prescribing practices?												
Yes	22 (96)	21 (91)	−0.33	0.56	26 (100)	26 (100)	-	-	48 (98)	47 (96)	−0.33	0.56
5. In the past 2 years, have you received any training in antibiotic prescribing and antibiotic resistance?												
Yes	0 (0)	6 (26)	20.0	**<0.001**	0 (0)	10 (38)	19.7	**<0.001**	0 (0)	16 (33)	33.0	**<0.001**

Bold = *p* value ≤ 0.05; * Results refer to those providing affirmative and/or correct responses.

**Table 5:** Number of responses indicating confidence in carrying out the prescribing activities included on the knowledge, beliefs, and practices questionnaire about Ni-Vanuatu health worker’s antibiotic prescribing, antibiotic resistance awareness, and the influence of the COVID-19 pandemic. The difference in mean confidence scores between the baseline (2018) and follow-up (2022) results are provided.

	Nurses, Midwives	Doctors, Dentists, Pharmacists		All Health Workers		
	*n* = 23	*n* = 26		*n* = 49		
Number of Responses Indicating Confidence in Prescribing Activities	Baseline *n* (%)	Follow-Up *n* (%)	Baseline *n* (%)	Follow-Up *n* (%)	Baseline *n* (%)	Follow-Up *n* (%)
0	0 (0)	1 (4)	0 (0)	0 (0)	0 (0)	1 (2)
1	0 (0)	1 (4)	0 (0)	0 (0)	0 (0)	1 (2)
2	0 (0)	0 (0)	0 (0)	1 (4)	0 (0)	0 (0)
3	0 (0)	1 (4)	0 (0)	1 (4)	0 (0)	3 (6)
4	2 (9)	1 (4)	1 (4)	2 (8)	3 (6)	3 (6)
5	0 (0)	1 (4)	1 (4)	1 (4)	1 (2)	2 (4)
6	2 (9)	6 (26)	3 (11)	3 (11)	5 (10)	9 (18)
7	5 (21)	2 (9)	5 (19)	3 (11)	10 (20)	5 (10)
8	14 (61)	10 (45)	16 (61)	15 (58)	30 (62)	25 (52)
Total number of possible affirmative responses = *n*	Nurses: *n* = 184		Doctors: *n* = 208		All health workers: *n* = 392	
Number and proportion affirmative = *n* (%)	167 (91)	143 (78)	190 (91)	177 (85)	357 (91)	320 (82)
Differences between mean confidence scores baseline and follow-up; *p* value (95% CI) *	−6.01; ***p* 0.02** (−7.39; −0.68)		−3.12; ***p* < 0.001** (−5.44; −1.63)		−3.77; ***p* < 0.001** (−1.97; −5.57)	

Bold = *p* value ≤ 0.05; * Paired T test.

**Table 6:** Number and proportion of respondents and their level of agreement with statements about antibiotic resistance (ABR) included on the knowledge, beliefs, and practices (KBP) questionnaire about Ni-Vanuatu health worker’s antibiotic prescribing and antibiotic resistance and the influence of the COVID-19 pandemic. The differences between the baseline (2018) and follow-up (2022) questionnaire results are provided.

Respondents		Nurses, Midwives			Doctors, Dentists, Pharmacists			All Health Staff		
		*n* = 23		WSRT *	*n* = 26		WSRT	*n* = 49		WSRT
		Baseline *n* (%)	Follow-Up *n* (%)	*p* Value	Baseline *n* (%)	Follow-Up *n* (%)	*p* Value	Baseline *n* (%)	Follow-Up *n* (%)	*p* Value
1. Antibiotic resistance (ABR) is a problem in the world.										
a.	Agree	19 (83)	21 (92)	0.20	23 (88)	18 (69)	0.42	42 (86)	45 (92)	0.11
b.	Neutral	2 (9)	1 (4)		2 (8)	6 (23)		4 (8)	3 (6)	
c.	Disagree	2 (9)	1 (4)		1 (4)	2 (7)		3 (6)	1 (2)	
2. ABR is a problem in PICTs.										
a.	Agree	14 (61)	18 (78)	0.17	19 (73)	13 (50)	0.44	33 (67)	41 (84)	0.04
b.	Neutral	8 (35)	4 (17)		6 (23)	10 (38)		14 (29)	7 (14)	
c.	Disagree	1 (4)	1 (4)		1 (4)	3 (12)		2 (4)	1 (2)	
3. ABR is a problem in Vanuatu.										
a.	Agree	19 (83)	17 (74)	0.62	16 (62)	22 (85)	0.21	35 (71)	39 (80)	**0.41**
b.	Neutral	3 (13)	4 (17)		7 (27)	3 (12)		10 (20)	7 (14)	
c.	Disagree	1 (4)	2 (9)		3 (12)	1 (4)		4 (8)	3 (6)	
4. Too many antibiotics being prescribed may contribute to ABR.										
a.	Agree	23 (100)	21 (92)	**0.001**	24 (92)	17 (65)	**0.004**	47 (96)	36 (78)	**0.001**
b.	Neutral	0	1 (4)		1 (4)	8 (31)		1 (2)	9 (18)	
c.	Disagree	0	1 (4)		1 (4)	1 (4)		1 (2)	2 (5)	
5. Patient’s antibiotic dose is too low may contribute to ABR.										
a.	Agree	10 (44)	12 (52)	0.93	13 (50)	18 (69)	0.64	23 (47)	30 (61)	0.79
b.	Neutral	6 (26)	8 (35)		11 (42)	7 (27)		17 (35)	15 (31)	
c.	Disagree	7 (30)	3 (13)		2 (8)	1 (4)		9 (18)	4 (8)	
6. Patients sharing antibiotics with family and friends may contribute to ABR.										
a.	Agree	19 (83)	20 (87)	0.22	24 (92)	25 (96)	**0.01**	43 (88)	46 (94)	**0.01**
b.	Neutral	0 (0)	3 (13)		0 (0)	1 (4)		0 (0)	1 (2)	
c.	Disagree	4 (17)	(0)		2 (8)	0 (0)		6 (12)	2 (4)	
7. Poor infection control in healthcare settings.										
a.	Agree	6 (26)	20 (87)	**0.01**	15 (58)	16(62)	0.15	21 (43)	42(86)	**0.003**
b.	Neutral	11 (48)	2 (9)		9 (35)	6 (23)		20 (41)	6(12)	
c.	Disagree	6 (26)	1 (4)		2 (8)	4 (15)		8 (16)	1(2)	
8. Health worker forgetting to perform hand hygiene when required.										
a.	Agree	19 (83)	19 (83)	**0.05**	15 (58)	18 (69)	0.96	34 (78)	37 (76)	0.25
b.	Neutral	0 (0)	1 (4)		6 (23)	5 (19)		6 (12)	6 (12)	
c.	Disagree	4 (17)	3 (13)		5 (19)	3 (12)		9 (18)	6 (12)	
9. High patient expectation to receive antibiotics.										
a.	Agree	23 (100)	19 (83)	**0.001**	24 (92)	22 (85)	**0.003**	47 (96)	41 (85)	**0.001**
b.	Neutral	0 (0)	3 (13)		2 (8)	4 (15)		2 (4)	7 (14)	
c.	Disagree	0 (0)	1 (4)		0	0 (0)		0 (0)	1 (2)	
10. Patients not completing their entire prescription.										
a.	Agree	21 (91)	20 (87)	0.13	25 (97)	22 (92)	**0.01**	46 (94)	44 (91)	**0.004**
b.	Neutral	0 (0)	2 (9)		0	4 (8)		0 (0)	4 (8)	
c.	Disagree	2 (9)	1 (4)		1 (4)	0 (0)		3 (4)	1 (2)	

* WSRT = Wilcoxon signed rank test; Bold = *p* ≤ 0.05.

**Table 7:** A comparison of participant responses to the open-ended question about factors that may prevent hand hygiene performance when required and are listed on the knowledge, beliefs, and practices (KBP) questionnaire about Ni-Vanuatu health workers’ KBP regarding antibiotic prescribing, antibiotic resistance, and the influence of the COVID-19 pandemic. The differences between baseline (2018) and follow-up (2022) questionnaire results are provided.

“Please List the Factors Which Make It Difficult or Prevent You from Performing Hand Hygiene When Required.”			
Theme	Baseline Response 2018	Follow-Up Response 2022	Interpretation
Lack of supplies and equipment	No soap and no alcohol hand rub. Supplies not replenished. No running water. Not enough places to wash hands in. Hand washing station out-of-order.	Problem of replenishing and acquiring sanitary supplies. Must buy own hand sanitiser now. Lack of equipment, e.g., gloves Only when there is no hand wash or soap. Water is usually available. No sink or tap in the consulting room. Not enough sinks around and soap dispenser empty	Both before and during the pandemic, issues with the procurement and distribution of antibiotic handwashing products prevented hospital staff from performing optimal hand hygiene.Pre-pandemic staff reported a lack of wash basins and those that were available were either not easily accessible or were out-of-order. During the pandemic there was a concern that washing facilities were not located within easy reach, preventing over-stretched health workers from performing hand hygiene whenever required.
Workload pressures	Too busy and forgetfulness. When in a rush. When I am interrupted to attend an urgent task while away from tap water.	Overcrowding and fatigue; cannot stop to wash hands. Emergency situations when immediate attention and hands-on is required. Bad timing; just forgot. In cases when there are too many patients, hand hygiene is not practiced.	Pre-pandemic responses suggest there were too few health workers on the ground to allow staff the opportunity to stop to perform hand hygiene. According to the follow-up responses, this scenario had not changed. The increased number of patients during COVID-19 combined with too few staff and staff fatigue may have left no time for consistent hand hygiene.

**Table 8:** Number and proportion of respondents and their level of agreement to statements and questions about prescribing practices included on the knowledge, beliefs, and practices (KBP) questionnaire about Ni-Vanuatu health workers’ KBP regarding antibiotic prescribing, antibiotic resistance, and the influence of the COVID-19 pandemic. The differences between baseline (2018) and follow-up (2022) questionnaire results are provided.

Respondents	Nurses, Midwives			Doctors, Dentists, Pharmacists			All Health Workers		
	*n* = 23		WSRT *	*n* = 26		WSRT	*n* = 49		WSRT
	Baseline *n* (%)	Follow-Up *n* (%)	*p* Value	Baseline *n* (%)	Follow-Up *n* (%)	*p* Value	Baseline *n* (%)	Follow-Up *n* (%)	*p* Value
1. Last month, how many times did you prescribe antibiotics?									
>1 time per day	10 (43)	13 (56)	0.98	13 (50)	12 (46)	0.38	23 (47)	25 (51)	0.73
3–5 times per week	7 (37)	1 (5)		9 (35)	9 (35)		16 (33)	10 (20)	
1–2 times per week	2 (9)	4 (17)		2 (10)	1 (4)		4 (8)	5 (10)	
>3 times per week	4 (17)	5 (22)		2 (10)	4 (15)		6 (12)	9 (18)	
2. How often are the antibiotics you need available?									
Always	8 (35)	14 (61)	**0.04**	11 (42)	9 (35)	0.34	19 (39)	23 (44)	**0.02**
Frequently	3 (13)	5 (22)		6 (23)	16 (62)		9 (18)	21 (43)	
Sometimes	12 (52)	4 (17)		9 (35)	1 (4)		21 (41)	5 (10)	
3. When I prescribe antibiotics, I use advice from a pharmacist.									
Always	10 (43)	5(22)	0.07	6 (23)	7 (27)	0.96	16 (33)	12 (24)	0.21
Sometimes	9 (39)	12(52)		13 (50)	12 (46)		22 (44)	24 (49)	
Never	4 (17)	6(26)		7 (27)	7 (27)		11 (22)	13 (27)	
4. When I prescribe antibiotics, I use bacterial culture results.									
Always	1 (4)	3(13)	**0.02**	10 (38)	5 (19)	0.16	11 (22)	8 (16)	0.68
Sometimes	10 (43)	15(65)		13 (50)	18 (69)		23 (43)	33 (67)	
Never	12 (52)	5(22)		3 (12)	3 (12)		15 (31)	8 (16)	
5. When I prescribe antibiotics, I use apps on a mobile device.									
Always	3 (13)	5(22)	0.23	(0)	6 (23)	**0.002**	3 (6)	11 (22)	**0.002**
Sometimes	5 (22)	9(39)		8 (31)	15 (58)		13 (27)	24 (49)	
Never	15 (65)	9(39)		18 (69)	5 (19)		33 (67)	14 (29)	
6. How many of your patients ask for antibiotics when antibiotics are not needed?									
Most	9 (39)	9 (39)	0.07	1 (4)	8 (31)	**0.04**	10 (20)	17 (35)	0.46
Some	13 (57)	6 (26)		14 (54)	10 (38)		27 (55)	16 (33)	
None	1 (4)	8 (35)		11 (42)	8 (31)		12 (25)	16 (33)	
7. Prescribing antibiotics when not required may cause harm.									
Agree	23 (100)	21 (91)	0.64	25 (96)	25 (96)	0.15	48 (98)	46 (94)	0.23
Undecided	(0)	1 (4)		1 (4)	1 (4)		1 (2)	2 (4)	
Disagree	(0)	1 (4)		(0)	(0)		(0)	1 (2)	
8. Prescribing antibiotics judiciously may reverse ABR.									
Agree	4 (17)	18 (78)	**0.007**	2 (8)	16 (62)	**0.01**	6 (12)	34 (69)	**0.001**
Undecided	17 (74)	3 (13)		24 (92)	6 (23)		41 (84)	9 (18)	
Disagree	2 (9)	2 (9)		(0)	4 (15)		2 (4)	6 (12)	
9. How often do you perform hand hygiene when required?									
Always	14 (61)	10 (43)	0.46	7 (27)	8 (31)	0.33	21 (43)	18 (37)	0.94
Sometimes	6 (26)	11 (48)		14 (54)	16 (62)		20 (35)	27 (55)	
Never	3 (13)	2 (7)		5 (19)	2 (7)		8 (16)	4 (8)	
10. How often do your colleagues perform hand hygiene when required?									
Always	4 (17)	9 (39)	**0.05**	4 (15)	5 (19)	0.14	8 (16)	14 (29)	**0.01**
Sometimes	8 (35)	8 (35)		8 (31)	13 (50)		16 (33)	21 (43)	
Never	11 (48)	6 (26)		14 (54)	8 (31)		25 (51)	14 (29)	
11. Able to identify from a list of 5 moments when hand hygiene is required to be performed **									
Yes	21 (91)	23 (100)	0.15	23 (88)	24 (92)	0.65	44 (90)	47 (96)	0.25
No	2 (9)	0(0)		3 (22)	2 (8)		5 (10)	2 (4)	

* Wilcoxon signed rank test; ** McNemar’s test; Bold = *p* ≤ 0.05.

**Table 9:** A representative selection of participant responses to the open-ended question, “Has the COVID-19 pandemic changed your antibiotic prescribing or awareness of antibiotic resistance (ABR) and in what way?” with interpretation.

	“Has the COVID-19 Pandemic Changed Your Antibiotic Prescribing or Awareness of ABR and in What Way?”.	
Theme	Qualitative Response	Interpretation
Thoughts about prescribing	I think COVID makes us forget about the importance of right dosage and right duration of antibiotics. We worry about the patient and do not want to get it wrong. Yes, we only give antibiotics if the patient has pneumonia, we have changed our practice, stricter. There is a need for more awareness around antibiotic prescription in relation to fever. Every patient who has a fever does not necessarily require antibiotics. Much the same. More reviewing the principles and guidelines of prescribing antibiotics. More awareness of what I prescribe because of inhouse training and more counselling during discharge. Yes, I think COVID changed our antibiotic prescribing as multiple awareness sessions tell us antibiotics will not help anyone with COVID-19.	This would suggest that health workers were overwhelmingly concerned about the wellbeing of their patients during COVID-19.There is a general consensus that since the pandemic started, health workers have become more careful about their prescribing practices. Pandemic preparedness training and treatment protocols accessible on mobile phones may have given health workers more confidence in not prescribing an antibiotic when one was not required. Health workers report reviewing their prescribing decisions with treatment guidelines from various sources.
Awareness of antibiotic resistance (ABR)	Sometimes I think more about antibiotic resistance than I did before—maybe because I am more aware of my prescribing decisions. Need to provide more advice these days on how medications can become resistant to certain drugs. More awareness of the dangers, taking too many antibiotics, not completing prescription, selling to others who cannot come to hospital or go to the health centre, or sharing antibiotics with family members. There should be more community awareness of ABR and taking antibiotics. People respond only to what health workers offer as best possible treatment; it has impact on antibiotic prescribing and consumption.	Health worker responses suggest that the pandemic may have increased their understanding of ABR and heightened their appreciation of the problems caused by ABR.There is a concern on the part of the respondents that their patients and the community as a whole need to become better informed about ABR. Responses also suggest health workers should spend more time counselling and advising their patients about their treatment choices and about the need to adhere to healthcare provider’s instructions.
Patient counselling	Greater concern for my patients in COVID times. Fewer patients come in and those who visit are sicker and need more of my attention. Yes…and more thorough during patient consultations. Need to provide more advice on how antibiotics can become resistant to certain drugs. Yes, I kindly ask my patients to use antibiotics more appropriately and I educate them on what is antibiotics and what it does for them. We do more consistent counselling in the clinic with our patients than before COVID. More concern for their wellness during COVID—some patients are very vulnerable. Well, for me, I am over-emphasising my counselling for viral infections now as they do not need to be treated with antibiotics.	Respondents’ reports being more aware of the vulnerability of the patients that visit the hospital since the pandemic commenced. These patients are “sicker”. Consultations are “more thorough” and “consistent” and health workers are spending more time counselling patients when an antibiotic is not needed than before the pandemic.There is a greater recognition about ABR amongst health workers who express the need to educate their patients about antibiotic use and resistance.

## Data Availability

The questionnaire can be obtained from the corresponding author.

## Supplementary Materials

The following Supplementary Materials can be downloaded at: https://www.mdpi.com/article/10.3390/tropicalmed8100477/s1, Table S1. Number and proportion of respondents at follow-up who selected the correct treatment options for the five additional clinical scenarios about antibiotic prescribing during COVID-19; Table S2. Statements used to assess the respondent’s level of confidence in performing the prescribing activities. Table S3. Number and proportion of respondents at follow-up who selected one or more of the reasons why patients ask for antibiotics when an antibiotic is not needed. Table S4. Number and proportion of respondents at follow-up who agreed with statements and questions about prescribing, antibiotic resistance, and training during the COVID pandemic.
