# Supplementary material for: The Impact of COVID-19 on Knowledge, Beliefs, and Practices of Ni-Vanuatu Health Workers Regarding Antibiotic Prescribing and Antibiotic Resistance, 2018 and 2022: A Mixed Methods Study
Source: Trop Med Infect Dis. 2023 Oct 18;8(10):477. doi: 10.3390/tropicalmed8100477 (PMC10611053; doi:10.3390/tropicalmed8100477)
Supplement: Supplementary file 1 [file tropicalmed-08-00477-s001.zip › tropicalmed-2593841-supplementary.pdf]

Supplementary Tables: S1, S2, S3 and S4.

Table S1. Number and proportion of respondents at follow-up who selected the correct treatment options for the five additional clinical scenarios about antibiotic prescribing during COVID-19 included on the knowledge, beliefs, and practices questionnaire about antibiotic prescribing, antibiotic resistance and the influence of COVID-19.

Respondents	**Nurses, Midwives**	**Doctors, Dentists, Pharmacists**	**All health-workers**
	N=23	N=26	N=49
	Follow-up n (%)	Follow-up n (%)	n (%)
What treatment would you recommend?			
1. A mother comes into emergency with her 4yr old who has fever (39^{0}C), coryzal nasal discharge and a sore throat for 3 days, otherwise well. The child tests negative for COVID-19.			
Yes	22 (96)	25 (97)	47 (97)
2. A 28yr old nurse vaccinated against COVID-19 experiencing no symptoms, spent approximately one hour without PPE in close contact with a person who tested positive to COVID-19 the following day.			
Yes	18 (78)	19 (73)	37 (72)
3. A 12yr old boy with a runny nose (clear fluid) and sore throat with no other symptoms presents at outpatient department with his mum.			
Yes	18 (78)	19 (73)	37 (72)
4. A 70yr old grandfather comes into emergency with breathing difficulties, muscle aches and pains and is in a confused state. He has an oxygen saturation rate of 89% on room air. He tests positive for COVID-19.			
Yes	22 (96)	23 (85)	45 (90)

Table S2. Statements used to assess respondent's level of confidence in performing the prescribing activities included on the knowledge, beliefs and practices questionnaire about Ni-Vanuatu health worker's antibiotic prescribing, antibiotic resistance and the influence of COVID-19 pandemic.

**Statement No.**	**Statement**
How confident are you: 1. *very confident*, 2. *confident*, 3. *undecided*, 4. *not confident*, 5. *very unsure* in performing the following activities?	
1	Discussing your treatment decisions with your patients.
2	Making a definitive diagnosis about bacterial sepsis/infection.
3	Not prescribing an antibiotic when the patient presents with no fever and no severity criteria and you are unsure of your diagnosis.
4	Choosing the correct dose, interval of administration
5	Using a combination therapy.
6	Switching from IV to PO.
7	Planning to streamline or stop antibiotic treatment according to clinical evolution and investigation
8	Selecting the most appropriate antibiotic when laboratory test results return positive for infection.

Table S3. Number and proportion of respondents at follow-up who selected one or more of the reasons why patients ask for antibiotics when an antibiotic is not needed included on the knowledge, beliefs and practices follow-up questionnaire about antibiotic prescribing, awareness of antibiotic resistance and the influence of COVID-19.

Respondents	All health workers N=49 n (%)
Why do you think patients ask for antibiotics when they may not need them? Select any that may apply.	
1. Patient anxiety due to COVID-19	13 (26)
2. Patient perceives antibiotics will make them better when they are sick.	42 (85)
3. Travel to hospital is a hardship so patient asks for antibiotics just in case.	38 (77)
4. Patient is unaware of risk associated with taking antibiotics when not needed.	37 (75)
5. Patient needs reassurance when an antibiotic is not needed.	27 (55)

Table S4. Number and proportion of respondents at follow-up who agreed with statements and questions about prescribing, antibiotic resistance (ABR) and training during the COVID pandemic listed on the knowledge, beliefs, and practices questionnaire about antibiotic prescribing, ABR and the influence of COVID-19.

	Nurses, Midwives	Doctors, Dentists, Pharmacists	All health-workers
	N=23	N=26	N=49
Respondents	Follow-up n (%)	Follow-up n(%)	n (%)
1. Since COVID commenced, do any of these scenarios apply?			
1.1 Fewer antibiotics are being prescribed.	7 (30)	9 (35)	16 (33)
1.2 Outpatients are given extra antibiotics, so they do not have to visit the hospital so often.	9 (37)	14 (54)	23 (47)
1.3 I think more about antibiotic resistance when I prescribe antibiotics	13 (57)	10 (38)	23 (47)
1.4 I spend more time now counselling my patients.	8 (35)	9 (35)	17 (35)
1.5 None of the above.	5 (22)	1 (4)	6 (12)
2. Are you confident in recognising that a patient may have COVID-19?			
Yes	15 (65)	18 (69)	33 (67)
3. Which of the following training sessions have you attended since the beginning of COVID-19?			
3.1 Correct hand hygiene procedures.	19 (83)	16 (62)	35 (71)
3.2 Donning and doffing PPE.	18 (78)	22 (85)	40 (82)

3.3 Triaging a suspected COVID patient.	11 (48)	9 (35)	20 (41)
3.4 Administering a COVID vaccine.	7 (30)	4 (15)	11 (22)
3.5 Collecting and taking pathology specimens for testing.	5 (22)	12 (46)	17 (35)
3.5 None of the above.	3 (13)	2 (8)	5 (10)
4. Has the COVID pandemic changed your antibiotic prescribing practice?			
Yes	16 (70)	22 (85)	38 (78)
